# Radiocesium mobility in different parts of the two major tree species in Fukushima

**DOI:** 10.1038/s41598-023-35852-8

**Published:** 2023-06-05

**Authors:** Takuya Manaka, Masatake G. Araki, Shinta Ohashi, Naohiro Imamura, Wataru Sakashita, Sumika Ogo, Masabumi Komatsu, Tadashi Sakata, Yoshiki Shinomiya

**Affiliations:** 1 Department of Forest Soils, Forestry and Forest Products Research Institute (FFPRI), Tsukuba, Ibaraki 305-8687 Japan; 2 Extension and Protection Division, Private Forest Department, Forestry Agency, Chiyoda, Tokyo, 100-8952 Japan; 3 Department of Wood Properties and Processing, FFPRI, Tsukuba, Ibaraki 305-8687 Japan; 4 Center for Forest Restoration and Radioecology, FFPRI, Tsukuba, Ibaraki 305-8687 Japan; 5 Hokkaido Research Center, FFPRI, Sapporo, Hokkaido 062-8516 Japan; 6 Department of Mushroom Science and Forest Microbiology, FFPRI, Tsukuba, Ibaraki 305-8687 Japan

**Keywords:** Biogeochemistry, Environmental sciences

## Abstract

Radiocesium (^137^Cs) released in the Fukushima Dai-ichi Nuclear Power Plant accident is still cycling in the forest ecosystem. We examined the mobility of ^137^Cs in the external parts—leaves/needles, branches, and bark—of the two major tree species in Fukushima, Japanese cedar (*Cryptomeria japonica*) and konara oak (*Quercus serrata*). This variable mobility will likely lead to spatial heterogeneity of ^137^Cs and difficulty in predicting its dynamics for decades. We conducted leaching experiments on these samples by using ultrapure water and ammonium acetate. In Japanese cedar, the ^137^Cs percentage leached from current-year needles was 26–45% (ultrapure water) and 27–60% (ammonium acetate)—similar to those from old needles and branches. In konara oak, the ^137^Cs percentage leached from leaves was 47–72% (ultrapure water) and 70–100% (ammonium acetate)—comparable to those from current-year and old branches. Relatively poor ^137^Cs mobility was observed in the outer bark of Japanese cedar and in organic layer samples from both species. Comparison of the results from corresponding parts revealed greater ^137^Cs mobility in konara oak than in Japanese cedar. We suggest that more active cycling of ^137^Cs occurs in konara oak.

## Introduction

The Fukushima Dai-ichi Nuclear Power Plant accident on 11 March 2011 released a large amount of radiocesium—especially ^137^Cs, which is of great concern because of its long physical half-life (30.17 years)^1,2^. Forest covers about 70% of the contaminated land area in Fukushima^3^, and forestry and wood production in this area has suffered serious damage^4^.

In the first few months after the accident, most of the ^137^Cs was trapped directly in the forest canopy—in evergreen coniferous stands in particular^5^. Monovalent cations of ^137^Cs are weakly and electrostatically bound to negatively charged sites of organic matter in the canopy—mainly leaves or needles^6–8^—but they are quickly washed out towards the forest floor by throughfall and then gradually transported via litterfall^9^. On the forest floor, ^137^Cs is preferentially and strongly fixed by clay minerals, mainly in the mineral soil layer^10,11^. More than 10 years have passed since the accident, and most of the deposited ^137^Cs is now retained in fixed form on the surface of the mineral soil layer^12^.

Nevertheless, ^137^Cs is still cycling in the forest ecosystem in quantities that are not negligible in the context of certain forest uses (e.g., there are strict criteria for the allowable activity concentration of ^137^Cs in general foods, including edible mushrooms). In addition to the foliar uptake of ^137^Cs that occurred in the canopy in the initial phase after the accident, before the washing out of ^137^Cs^13^, part of the ^137^Cs in the forest soils was taken up by tree roots in competition with nutrients—mainly monovalent cations of K^14–16^. The internal translocation of ^137^Cs in trees is complicated: not only does the ^137^Cs activity concentration vary in different parts such as leaves/needles and sapwood^17,18^, the translocation processes to, and affinities for, each plant tissue also vary^19–22^. In addition, ^137^Cs translocated to the external parts of trees—leaves/needles, branches, and bark, which were the main targets of this study—is partly leached by rainwater (via throughfall or stemflow) and then transported again to the forest floor in a bioavailable form^12,23–26^. The differences in the internal structure, chemical composition, and morphology of these tree parts^27,28^ should lead to different ^137^Cs percentages leached from the parts. The lifespans and degradability of the different tree parts should also differ, leading to the existence of organic matter of various types and degrees of degradation on the forest floor. All of these differences result in spatial heterogeneity of ^137^Cs in forest ecosystems. Thus, long-term investigations at multiple sites with a large sample size are required for forecasting ^137^Cs dynamics in forest ecosystems in future decades^12,26,29,30^.

Here, we focused on ^137^Cs cycling in stands of the two major tree species in Fukushima, namely Japanese cedar (*Cryptomeria japonica*) and konara oak (*Quercus serrata*). The former is a major evergreen coniferous tree planted and used mainly for timber, whereas the latter is a deciduous broadleaf tree that often forms a secondary forest in this area and is used mainly for as logs for mushroom cultivation^4^. Stands of both species are often found in relatively close proximity, and they therefore suffered from radiocesium contamination of the same order of magnitude (e.g., at our sampling sites, shown in the following section). However, in previous studies conducted in the initial phase after the accident, ^137^Cs cycling was considered to be less dynamic in stands of konara oak than in Japanese cedar; because the accident occurred before the flush of shoots on deciduous trees, and the larger part of ^137^Cs falling on konara oak stands was deposited directly on the forest floor and more quickly fixed by clay minerals^5,24^. However, ^137^Cs contamination of the inner parts (such as the sapwood) of konara oak is still a severe problem^18^. It is therefore important to compare and clarify ^137^Cs movement in these two species.

In both species, we conducted leaching experiments on not only different external parts but also fallen leaves/needles and organic layer samples, to examine the mobility of ^137^Cs that had already been taken up and translocated to these parts. Although similar leaching experiments have been conducted on leaves/needles as well as decomposing litters in the context of the chemical forms of ^137^Cs or its downstream transport, and the differences in ^137^Cs mobility in each sample have been reported [e.g., Hara et al. (2020) and Kurihara et al. (2020)^31,32^], as far as we know no study has yet focused on ^137^Cs mobility and its variations in various external parts of trees. As leaching solutions, we adopt ultrapure water and ammonium acetate to create analogues of ^137^Cs mobility on two levels. Leaching with the former solution represents leaching by rainwater, targeting substantially mobile ^137^Cs and its active recirculation in the forest ecosystem. In contrast, leaching with the latter solution is often used in the field of soil science as an analog of the stock of bioavailable nutrients: cations that was electrostatically and temporarily bound to negatively charged sites on the surface of organic matter or clay minerals^33^. In our experiments, we expected that the ammonium acetate solution would leach ^137^Cs from the samples more efficiently than ultrapure water, through processes of cation exchange with excess ammonium. Furthermore, in some samples, leaching of other alkali metals (K, Rb, and the stable isotope ^133^Cs) was also evaluated, in the context of competition with ^137^Cs.

To recapitulate, the purposes of this study were (1) to assess ^137^Cs mobility in different external parts of trees; and (2) to compare current ^137^Cs cycling between Japanese cedar and konara oak stands; through leaching experiments. We expected that our experiments would reveal the differences in ^137^Cs mobility in different external tree parts or in different tree species, thus providing some biochemical insights into spatially heterogeneous ^137^Cs cycling in the forest ecosystem and its forecasting in the future.

## Materials and methods

### Materials

**Figure 1:**
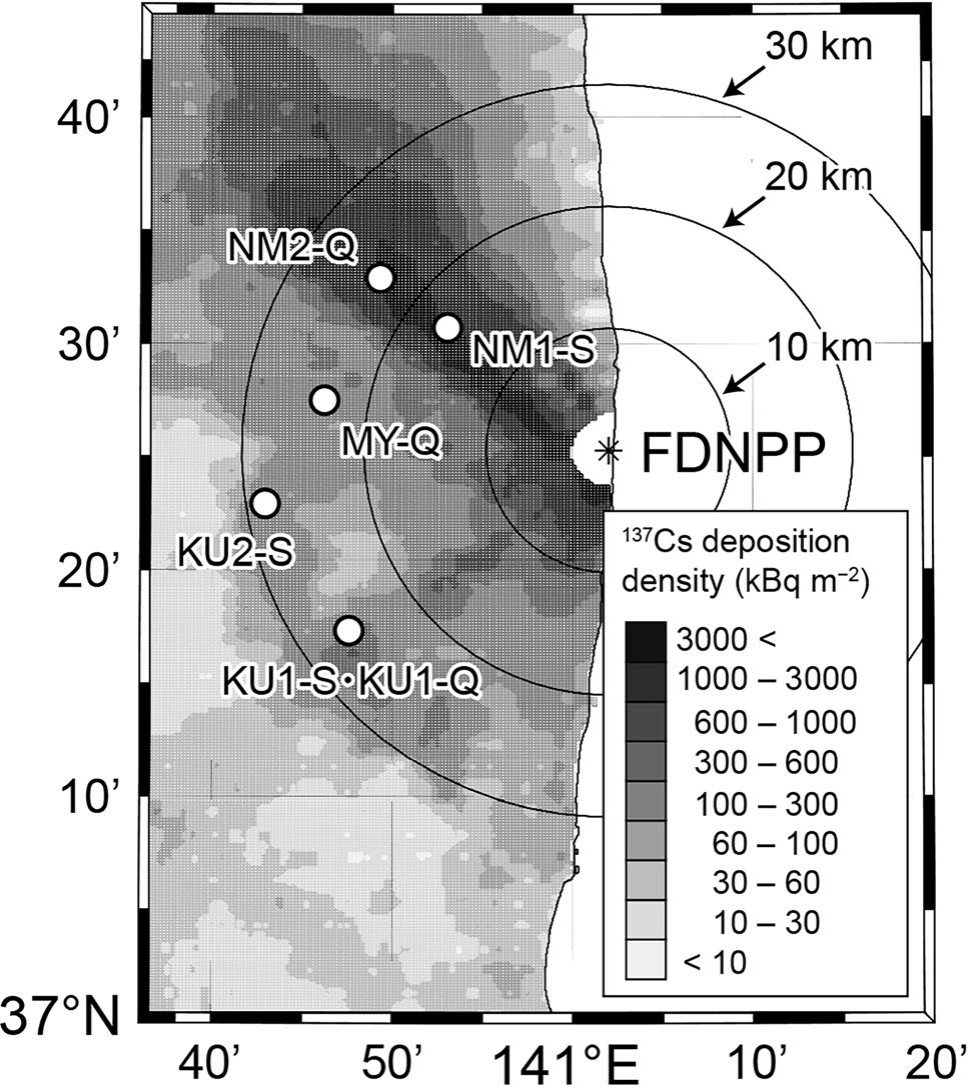
Locations of our six study sites. The sites are shown on a distribution map of ^137^Cs in Fukushima, on 28 June 2012, obtained from the fifth airborne monitoring survey data^36^. We also used MATLAB and M_Map^65^ to generate the map.

We used various external tree parts that had direct contact with rainwater: leaves/needles, branches, and bark from Japanese cedar and konara oak stands in the middle to mature stage (stand age: 26–80 years in 2011) in Fukushima, as well as fallen leaves/needles and organic soil layer samples. We established six sampling plots in total, and these two species were identified by authors at each plot. The sampling surveys were conducted in 2014 and 2021–2022, when most of the initially canopy-intercepted ^137^Cs had already been transferred to the forest floor via throughfall and litterfall^9,34^. For all sample collection, we avoided the flushing stage (May–June)^17,35^, when translocation of ^137^Cs among various tree organs was considered to be active. These data are summarized in Table 1 and Fig. 1. Note that “S” and “Q” in the plot names indicate the dominant species in the plot, namely Japanese cedar (“Sugi” in Japanese) and konara oak (*Quercus*), respectively. The distance between plots KU1-S and KU1-Q was about 500 m; initial ^137^Cs deposition in these two areas was of the same order of magnitude^36^.

**Table 1:** General characteristics of the sampling plots. The deposition data for ^137^Cs at each plot were obtained from the fifth airborne monitoring survey data collected on 28 June 2012^36^.

Dominant species	Site	Latitude	Longitude	Distance from FDNPP (km)	Elevation (m)	^137^Cs deposition (kBq m^−2^)	Stand age in 2011 (year)
Japanese cedar (*Cryptomeria japonica*)	KU1-S	37°17′18"N	140°47′48"E	26	660	630	43
	KU2-S	37°22′53"N	140°42′58"E	28	690	160	55
	NM1-S	37°30′40"N	140°53′05"E	17	140	3,800	38
Konara oak (*Quercus serrata*)	KU1-Q	37°17′22"N	140°47′30"E	27	720	470	26
	NM2-Q	37°32′54"N	140°49′20"E	24	490	3,300	80
	MY-Q	37°27′28"N	140°46′14"E	24	500	240	Not available

**Figure 2:**
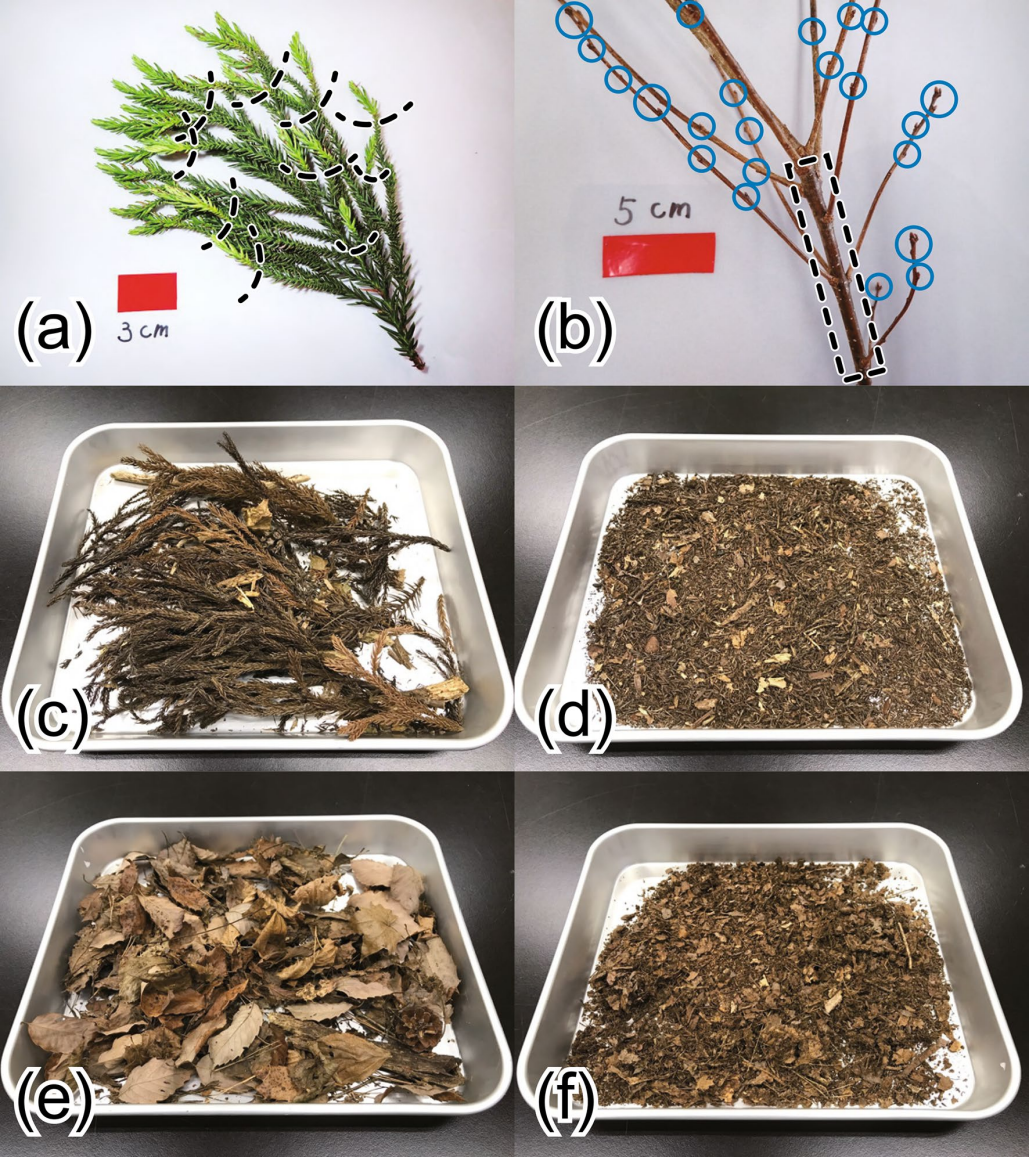
Photos of parts in which we examined the mobility of ^137^Cs. (**a**) Shoots of Japanese cedar in summer. The dashed black lines indicate the wave of growth, where the sample was divided into current-year and old needles. (**b**) Shoots of konara oak in spring. On the basis of the locations of the terminal bud scale scars, we distinguished current-year and old branches outside and inside the dashed black dashed box, respectively. Lateral and terminal buds at this time of year, which can be used as markers for sampling current-year branches, are shown in blue open circles. (**c**) Oi and (**d**) Oe and Oa layer samples from Japanese cedar stands, and (**e**) Oi and (**f**) Oe and Oa layers samples from konara oak stands, air-drying in aluminum trays (external dimensions: L 370 mm × W 307 mm × H 45 mm). These photos were taken before the samples were oven dried and roughly crushed.

Living samples of leaves/needles were collected directly from the canopy at plots KU1-S, KU1-Q, and KU2-S by using pruning shears. For Japanese cedar, the samples were separated into current-year and more-than-1-year-old needles (defined here as old needles) (Fig. 2a). For needles of Japanese cedar collected in 2021, to examine the variations in ^137^Cs mobility at different heights within an individual tree, the crown of each tree was bisected visually into upper and lower layers, and the needles were collected separately (Table S1).

Living branches were also collected directly from the canopy. For konara oak, the samples were separated into current-year and old branches (Fig. 2b). Although some konara oaks sprout two or three times in a year, we did not differentiate these but instead examined all current-year branches as a whole. In the case of Japanese cedar, it is difficult to distinguish strictly between needles and branches (Fig. 2a). We therefore collected branch samples that were clearly identifiable as branches (e.g., brown and without leaves). Branches of various thicknesses were collected in approximately equal amounts, and their ages were not considered. In 2021, branches, like needles, were collected from different heights within individual trees (Table S1).

Our bark samples of Japanese cedar from plots KU1-S and KU2-S were separated into two categories: outer and inner bark. Bark samples at 1.3 m above the ground (breast height) were collected directly from the tree stems with chisels, whereas samples at 5, 10, and 15 m above the ground were collected from stem disks after the trees had been felled.

Fallen leaves/needles were collected at plots NM1-S and NM2-Q by using funnel-shaped litter traps with an area of 0.5 m^2^ set at 1 m height during a period of about one month in autumn (from 13 October to 22 November 2021). After collection, the samples were washed quickly with ultrapure water to remove attached dust and debris, and then air dried. We also collected the organic layer in the vicinity of the litter traps. These samples were collected from the Oi layer and combined Oe and Oa layers, which are differentiated by the decomposition level of the plant debris (Fig. 2c–f).

Although voucher specimens are not deposited in a publicly available herbarium, all samples are archived in FFPRI.

### Leaching experiments

Leaching experiments were conducted in the following settings, modified from the work of Manaka *et al*. (2019, 2021)^25,37^; all samples were oven dried at 70–75 °C and then crushed roughly to a diameter of < 1 cm by using scissors or a cutting mill (U-140, Horai Co. Ltd., Higashi Osaka City, Osaka, Japan; or WT-150, Irie Shokai, Chiyoda City, Tokyo, Japan). Each sample and the leaching solutions [ultrapure water or ammonium acetate (1 mol kg^−1^, pH 7.0)] was placed in a polypropylene centrifuge tube at a weight ratio of 1:20, and mixed for 2 h by a reciprocating shaker at room temperature. Then, the mixture was immediately centrifuged, and the supernatant solution was passed through a 0.45-μm-pore-size filter (e.g., Dismic 25HP045AN, Advantec Toyo Kaisha, Ltd., Chiyoda City, Tokyo, Japan) and stored under cool and dark conditions.

### Radiocesium analysis

The pre-leaching samples and the filtrates with ultrapure water or ammonium acetate were placed separately into containers (100-mL U8 containers made of polypropylene or polystyrene, or 700- or 2000-mL Marinelli beakers made of acrylic resin) to measure ^137^Cs radioactivity. We used HPGe coaxial and P-type reverse coaxial detector systems (GEM20-70, GEM40P4-76, GEM-FX7025P4-ST, and GWL-120–15-LB-AWT, Ortec, Oak Ridge, TN, USA) and spectral analysis software (DS-P1001 Gamma Station, Seiko EG&G Co., Ltd., Chuo City, Tokyo, Japan). The measurement systems were calibrated regularly by using a standard gamma-ray source (MX033U8PP, Japan Radioisotope Association, Bunkyo City, Tokyo, Japan), and the reference standard material IAEA-444 was used to verify the accuracy. Measurement was continued until the statistical counting error became < 10% of the measured activity or the measurement time exceeded 86,400 s. For each part of the trees, the ^137^Cs activity concentrations (unit: Bq kg^−1^) were decay corrected to the representative day of sampling (Table S1).

### Potassium, rubidium, and stable cesium analysis

In the case of some samples of leaves/needles and branches, an aliquot of the filtrate with ultrapure water or ammonium acetate was diluted with 2% nitric acid, and the concentrations of K, Rb, and ^133^Cs were measured via ICP-MS (Agilent7700X; Agilent Technologies, Santa Clara, CA, USA). The relative standard deviation values for this measurement were approximately 5%. The quantitation limit for ^133^Cs was 25 ng kg^−1^ (at the time of sample introduction to the device), as estimated from repeated measurements of the calibration blank (2% nitric acid).

For these samples, we also analyzed the initial concentrations of K, Rb, and ^133^Cs before the leaching experiments. An aliquot of each pre-leaching sample was digested by using the wet ashing method with nitric acid and hydrogen peroxide in a heating block system (DigiPREP Jr.; SCP Science, Baie D’Urfé, Québec, Canada). The digested sample was passed through a 0.45-μm-pore-size filter, and the filtrates were analyzed by ICP-MS as described above.

### Data analysis

The percentage of leached ^137^Cs was calculated as follows:

1 $${\text{Percentage of leached }}^{{{137}}} {\text{Cs }} = \, \left( {\left[ {^{{{137}}} {\text{Cs}}} \right]_{filtrate} M_{solution} } \right)/ \, \left( {\left[ {^{{{137}}} {\text{Cs}}} \right]_{initial} M_{sample} } \right) \, \times { 1}00 = \, \left[ {^{{{137}}} {\text{Cs}}} \right]_{leached} / \, \left[ {^{{{137}}} {\text{Cs}}} \right]_{initial} \times { 1}00$$

where [^137^Cs]_*filtrate*_ is the activity concentration of ^137^Cs in the filtrate, and [^137^Cs]_*initial*_ is the initial activity concentration of ^137^Cs in the sample before leaching experiments (unit: Bq kg^−1^). The *M*_*solution*_ and *M*_*sample*_ value indicate mass of leaching solution (ultrapure water or ammonium acetate) and sample used in the experiments, respectively (unit: kg). In consideration of the experiments conducted in a weight ratio of 1:20, *M*_*solution*_ is equal to 20 times *M*_*sample*_, and we redefine [^137^Cs]_*leached*_ as ([^137^Cs]_*filtrates*_ × 20), the activity concentration of leachable ^137^Cs from the sample.

Some samples had higher [^137^Cs]_*leached*_ values than [^137^Cs]_*initial*_, because of sample heterogeneity or analytical error, or both. In these cases we therefore set [^137^Cs]_*leached*_ / [^137^Cs]_*initial*_ to 1 (i.e. 100%). Similar calculations were conducted for K, Rb, and ^133^Cs.

The mobility of ^137^Cs in different external parts of the two major tree species were compared by R version 4.0.3. First, the [^137^Cs]_*leached*_ / [^137^Cs]_*initial*_ values for each leaching solution were logit transformed as

2 $$p = {\text{ log }}(\left( {\left[ {^{{{137}}} {\text{Cs}}} \right]_{leached} / \, \left[ {^{{{137}}} {\text{Cs}}} \right]_{initial} + \varepsilon } \right) \, / \, \left( {{1 }{-} \, \left[ {^{{{137}}} {\text{Cs}}} \right]_{leached} / \, \left[ {^{{{137}}} {\text{Cs}}} \right]_{initial} + \varepsilon } \right)$$

One difficulty with the logit transformation was that in our experiments the precondition [^137^Cs]_*leached*_ / [^137^Cs]_*initial*_ values could be equal to 0 (i.e. 0%) or 1 (i.e. 100%). In such cases, this equation was transformed to undefined values of infinity. To prevent this, an arbitrary small value of *ε* was added to both the numerator and denominator^38^. Considering that the smallest non-zero observed percentage in our previous study^25^ was 0.5%, we adopted a *ε* value of 0.005. We performed a linear regression analysis to assess *p* values in comparisons of different parts. Multiple comparisons by using Tukey’s test were performed, with the glht function of the R multicomp package.

For ^137^Cs leaching percentage of needles and branches of Japanese cedar, similar multiple comparisons were performed at different relative crown positions. In contrast, linear regression analysis was performed between the percentage of ^137^Cs leaching from bark in Japanese cedar and bark sampling height above the ground.

## Results

**Figure 3:**
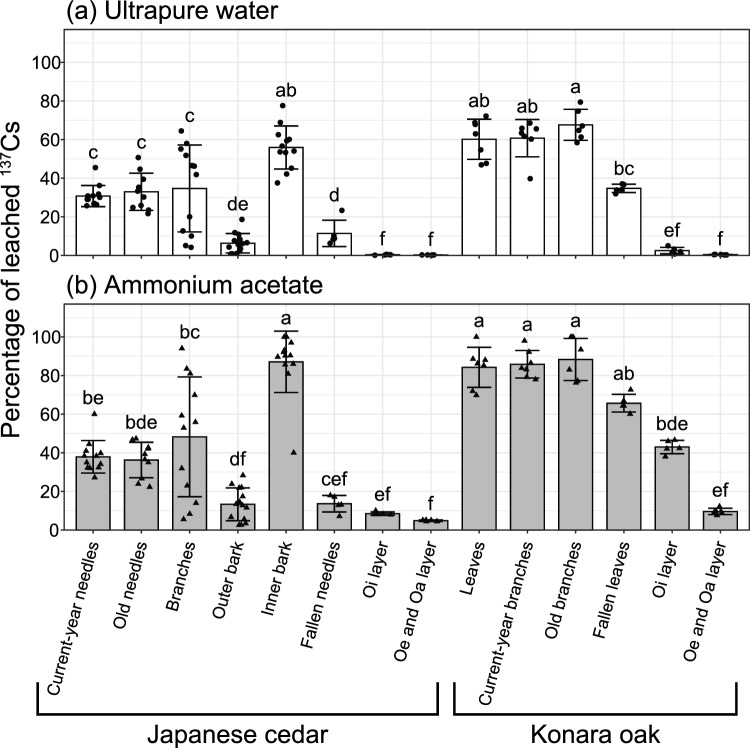
Bar charts of mean percentages of ^137^Cs leached in (**a**) ultrapure water and (**b**) ammonium acetate from different parts of the two major tree species. Error bars indicate one standard deviation (SD). Note that samples of each part from different sampling plots, years, and positions or heights (only for leaves/needles, branches, and bark) were lumped together. For each leaching solution, different letters indicate significant differences among the parts (*P* < 0.05).

Percentage of leached ^137^Cs by ultrapure water or ammonium acetate from our samples were summarized in Table S1 and Fig. 3. For the same sample, the leaching percentage by ultrapure water [e.g., 26–45% (average 31%) for current-year needles from Japanese cedars] was overall smaller than that of ammonium acetate [27–60% (average 38%)].

The percentages of ^137^Cs leached by water or ammonium acetate were similar for needles (both current-year and old ones) and branches from Japanese cedar (Table S1 and Fig. 3). We observed relatively large variations in leaching percentages from Japanese cedar branches—even those collected from the same plot [e.g., 4.2–65% (average 35%)]—in ultrapure water. The percentages leached from the inner bark of Japanese cedar were high [e.g., 38–78% (average 56%) in ultrapure water], whereas those from the outer bark were very low [0.70–19% (average 6.3%) in ultrapure water]. In konara oak, the percentages leached from leaves and branches (both current-year and old ones) were also similar. In contrast, in both species, the percentages leached from organic layer samples were extremely low [e.g., in ultrapure water, 0.19–0.54% (average 0.35%) from the Oi layer and 0.16–0.29% (average 0.25%) from the Oe and Oa layers under Japanese cedar]; the next highest were for fallen leaves/needles, followed by ones still attached to the tree. These differences were statistically examined in our multiple comparisons (Fig. 3).

**Figure 4:**
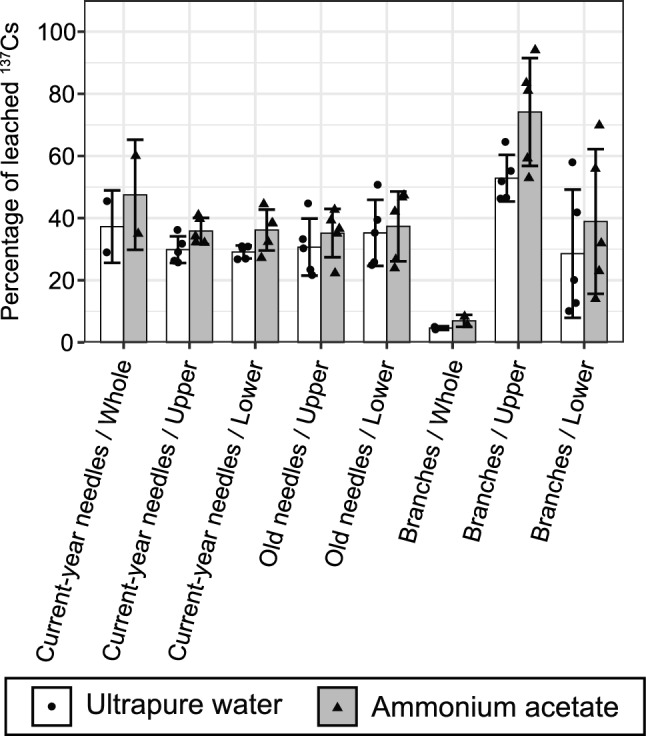
Bar chart of mean percentages of ^137^Cs leached in ultrapure water and ammonium acetate from current-year and old needles, and branches, in different positions on Japanese cedar. The error bars indicate one standard deviation (SD). Except in the case of samples from “whole” positions, of which we had few, we performed multiple comparisons for each leaching solution. However, significant differences (*P* < 0.05) were observed only between branches from the “upper” position and samples of all other parts and positions.

**Figure 5:**
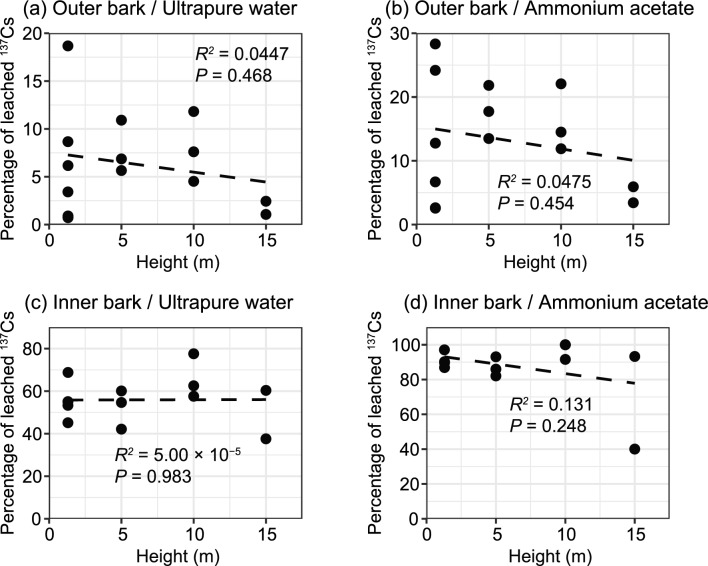
Scatter plots of percentage of leached ^137^Cs and sampling height (m) of bark of Japanese cedar. Results are shown for outer bark in (**a**) ultrapure water and (**b**) ammonium acetate, and for inner bark in (**c**) ultrapure water and (**d**) ammonium acetate. Linear regression analyses were performed for all samples, but significant trends (*P* < 0.05) were not observed for any part or with either leaching solution. Regression lines are shown as dashed lines.

In 2021, needles, branches, and bark of Japanese cedar were collected from different relative crown positions or heights. We compared their percentages of ^137^Cs leached by using multiple comparisons and linear regression analysis. For most tree parts, significant differences or trends were not observed between different heights or crown positions (Table S1 and Figs. 4 and 5).

Comparison of variations between the two species revealed that the corresponding parts of konara oak (e.g., leaves/needles and branches) had higher leaching percentages than those of Japanese cedar (Table S1 and Fig. 3).

**Figure 6:**
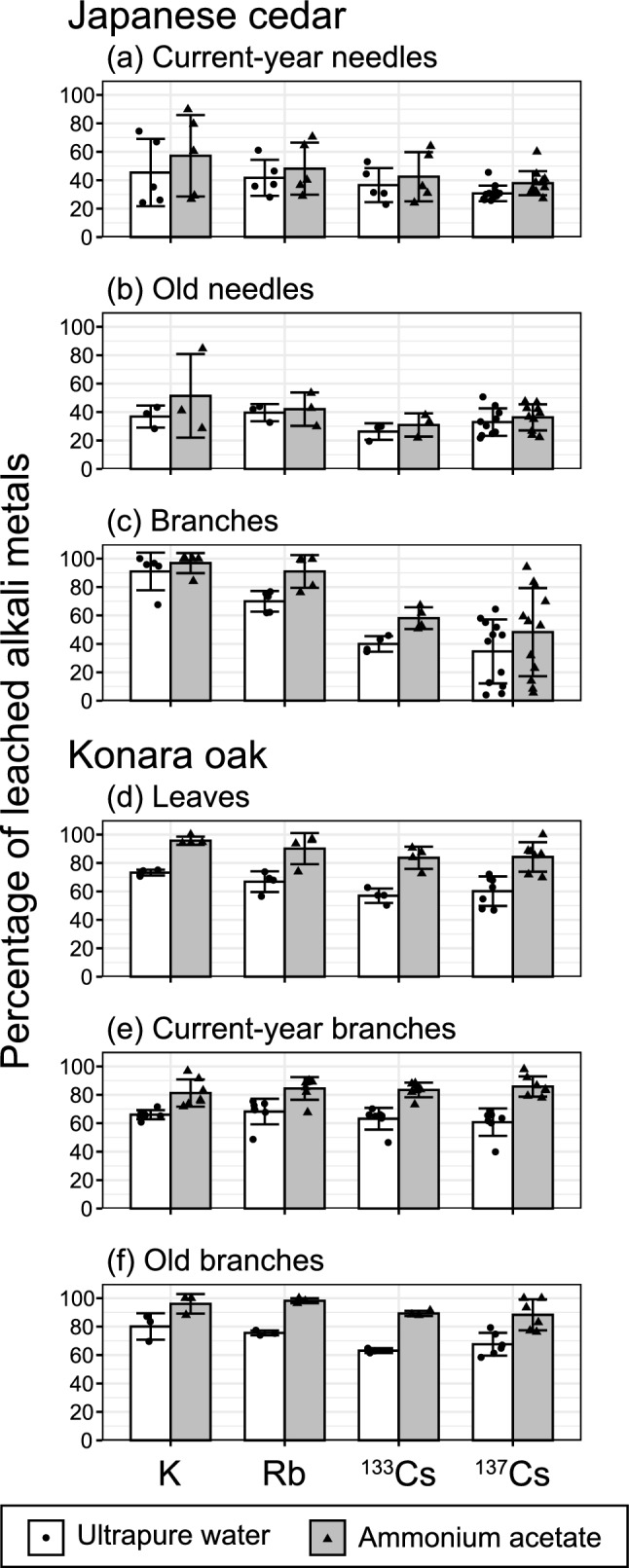
Bar charts of mean percentages of alkali metals (K, Rb, ^133^Cs, and ^137^Cs) leached in ultrapure water and ammonium acetate from (**a**) current-year needles, (**b**) old needles, and (**c**) branches of Japanese cedar; and (**d**) leaves, (**e**) current-year branches, and (**f**) old branches of konara oak. Samples marked “N.D.” in Table S1 for the percentage of ^133^Cs leached have been omitted. Error bars indicate one standard deviation (SD). Note that samples of each part from different sampling plots, years, and positions were lumped together. Multiple comparisons were not performed because of the small sample sizes in the cases of K, Rb, and ^133^Cs.

We plotted the mean percentages of K, Rb, and ^133^Cs, as well as ^137^Cs, leached by ultrapure water or ammonium acetate from our samples (Table S1 and Fig. 6). Unfortunately, multiple comparisons were not performed because of the small sample size (*n* = 3 at minimum). However, in particular for branches of Japanese cedar, leaching percentages of K tended to be higher than those of other alkali metals [68–100% (average 91%) in ultrapure water, and 84–100% (average 97%) in ammonium acetate]. In these samples, the leaching percentages of ^133^Cs [35–46% (average 40%) in ultrapure water and 51–67% (average 58%) in ammonium acetate, excluding samples showing “N.D.”] were comparable to those of ^137^Cs.

## Discussion

### Difference in ^137^Cs mobility among external tree parts

Plant tissues and soil organic matter generally possess negatively charged sites such as carboxylic and hydroxyl groups. Monovalent cations of ^137^Cs are weakly and electrostatically bound to these sites, forming outer-sphere complexes, and they can be easily replaced by ubiquitous cations such as K and Ca in rain and soil water and washed out^6–8^. In our leaching experiments, excess ammonium in ammonium acetate was expected to efficiently replace ^137^Cs, as well as other alkali metals, in the samples ^33^. However, first, we highlight the finding that some of the ^137^Cs could not be leached out, even by this solution (Table S1 and Fig. 3). For example, about 60% of the ^137^Cs was not leached from current-year needles of Japanese cedar. A possible explanation for this result is that some ^137^Cs is physically trapped in the interior matrix of the plant tissue; one contributor to this trapping is the complex nature of the structure of persistent organic matter components such as lignin. This possibility is also suggested by the results of previous decomposition or extraction experiments using actual plant parts and soil organic matter^19,30,31,39^. Some of these results will be compared with our results in the discussion below.

Our leaching experiments revealed various degrees of mobility of ^137^Cs in each type of external tree part (Table S1 and Fig. 3). First, current-year and old needles of Japanese cedar had statistically similar ^137^Cs leaching percentages. In addition, significant leaching differences were not observed between needles sampled from different relative crown positions (Fig. 4). In the case of old needles, direct ^137^Cs contamination of the needle tissues were reported in the initial phase after the Fukushima accident^5,13^. However, our results suggest that this effect might have already become small in our sampling period—even in 2014; that is, replacement of the initially contaminated needles with new needles had already begun to occur^9,34^, and ^137^Cs was being evenly translocated and cycled between the new and old needles.

The percentage of ^137^Cs leached from branches of Japanese cedar was similar to the percentages leached from current-year and old needles (Table S1 and Fig. 3). Similar results were also shown in konara oak. Notably, we observed markedly large variations in leaching percentages among Japanese cedar branches—even in samples from the same plot. The exact reason for this variation is unclear, but it might be related to the complex physiological structure of the branch tissues of this species. Although we collected branch samples that were clearly identifiable as branches, not only did the lengths and diameters of the samples vary but also the proportions of bark (which had extremely low ^137^Cs mobility, as discussed in the next paragraph) and pith. These differences may explain the large variations.

In the case of Japanese cedar, the percentages leached from outer bark were much lower than those from inner bark and from needles and branches (Table S1 and Fig. 3). A possible explanation is that the rough surfaces on the outer bark effectively captured insoluble particles bearing ^137^Cs^40^. In addition, some of these particles might have been derived from the initial phase after the accident. Although very few studies have focused directly on the lifespan of outer bark, it is likely to be longer than that of needles, so that the impact of the initial contamination might have remained in our bark samples. Imamura et al. (2017)^41^ reported that the activity concentration of ^137^Cs of bark was higher than those of leaves/needles, branches, and wood as of 2015, and on the basis of five-year monitoring after the accident, they suggested that ^137^Cs had a longer residence time in the outer bark. During this long residence time, the remaining leachable fraction of ^137^Cs in the outer bark^40^ might have been washed away by stemflow. Furthermore, previous studies have suggested that the outer bark has higher sorption capacity for soluble metal ions, including ^137^Cs^37,42^. Thus, even small input of outer bark to the forest floor might lead large spatial heterogeneity of ^137^Cs activity concentrations and bioavailability there. Incidentally, the complex morphology and water storage capacity of bark can generate spatial variations in ^137^Cs activity concentrations within the bark, as well as spatial variations in ^137^Cs outflow via stemflow^26,43^. This variation might have led to the lack of significant differences in leaching percentages between our bark samples taken at different heights (Fig. 5).

The percentages of ^137^Cs leached from fallen needles of Japanese cedar were smaller than those from still attached needles, and a similar trend was observed in the leaves of konara oak, but these differences were significant only in Japanese cedar and in the case of ultrapure water (Table S1 and Fig. 3). Reabsorption of mobile ^137^Cs from senesced leaves/needles into the tree body occurs before defoliation^17,35^, and might have been the reason for the above finding. In contrast, in both species, the organic layer samples had extremely small leaching percentages—close to zero in the case of ultrapure water. A possible explanation for these small values is that the leachable fraction of ^137^Cs in the original litter had already been flushed out^32,44^, and the surrounding clay minerals preferentially and strongly fixed the ^137^Cs^10,11^. In addition, it has been suggested that ^137^Cs accumulates and is immobilized in persistent soil organic matter and aggregates with minerals^30,39,45^, and, further, that soil microorganisms contribute to this fixation^46^.

Leaching experiments on leaves/needles and on fallen leaves/needles and decomposing litter have been conducted in previous studies, although the focus of some of these studies differed from ours (e.g., ^137^Cs downstream transport). We therefore compared our results and our experimental conditions with findings of these other studies (Table S2). Hara et al. (2020)^31^ reported much higher percentages of ^137^Cs leached from current-year needles of Japanese cedar (represented as “2015 segment” in Table S2: 96.8% for water-soluble ^137^Cs) than our study found. The exact reason for this difference is unclear: perhaps, in their study, sampling in the flushing stage (June) and from a relatively low height (at 4 m from trees with an average height of 22.7 m), and the difference in pulverizing treatments (to a much finer level than ours) before extraction, might have led to the elution of abundant mobile ^137^Cs. In contrast, the percentages of ^137^Cs leached from fallen leaves/needles in other studies^47–49^ were comparable to ours. For example, the results (e.g., 44.2% for soluble ^137^Cs), as well as the experimental conditions (e.g., a weight ratio of 1:20 and 0.5 h of shaking) for fallen deciduous broadleaves in the work of Saito et al.^49^ were similar to ours. The results of experimental leaching from decomposing litter in previous studies^25,32,44,49–51^ were also comparable to ours: extremely small leaching percentages—in particular in water—were reported.

It is generally considered that K act as a competitor of ^137^Cs through uptake processes in forest soils^14,16,52^. In contrast, some of the studies listed in Table S2 also examined the leaching and extraction of other alkali metals: Hara et al. (2020)^31^ in needles of Japanese cedar, Sakai et al. (2015)^47^ in fallen needles of Japanese cedar, and Kurihara et al. (2020)^32^ in the organic layer under both species. All of them reported significantly higher mobility of K than of ^137^Cs. As shown in our Results and in Table S1 and Fig. 6, although we did not perform multiple comparisons of the leaching percentages of alkali metals, we also observed a trend toward higher mobility of K and Rb in some tree parts. Although the exact reason for different mobility of alkali metals are unknown and additional studies are needed, not only different ionic radii and hydration energy of these ions, but also different affinities for plant tissues and translocation system^17,20,21^ likely affect the different mobility. Moreover, our results showed similar trends in mobility between ^137^ and ^133^Cs. These results might indicate that ^137^Cs dispersed by the accident is reaching equilibrium with ^133^Cs—an ambient stable isotope of Cs in the natural environment—in the tree biomass^15^.

### Differences in ^137^Cs between species

Comparison of leaching percentages in the leaves/needles and branches revealed that Konara oak had generally greater ^137^Cs mobility than Japanese cedar (Table S1 and Fig. 3). We therefore suggest that more active cycling of ^137^Cs occurs in konara oak.

The accident in 2011 occurred in March, before the flush of shoots on deciduous trees. Whereas the needles and branches of Japanese cedar were directly contaminated with ^137^Cs, in the konara oak stands a large proportion of the ^137^Cs was deposited directly on the forest floor^5^. In addition, the decomposition rate of deciduous broadleaves is generally faster than that of coniferous needles^53^. For these reasons, from an analysis of monitoring data obtained in the first few years after the accident, Koarashi et al. (2016)^24^ suggested that ^137^Cs would have been transferred to the mineral soil layers and irreversibly fixed there more quickly under konara oak than under Japanese cedar; therefore, ^137^Cs cycling in the forest ecosystem would have been less active in the case of konara oak.

However, much longer monitoring and modeling analyses in forests with the same initial deposition levels of ^137^Cs have revealed that, the activity concentration of ^137^Cs is higher even in the inner tree parts, such as the sapwood, of konara oak than in Japanese cedar [e.g., Ohashi et al. (2022)^18^; they analyzed samples from plots KU1-S and KU1-Q]. Moreover, the increasing trends in this activity concentration in konara oak have been estimated to continue much longer^54^.

The uptake of ^137^Cs from soil to trees is often evaluated by using an aggregated transfer factor, namely, the ratio of the ^137^Cs activity concentration in each tree part to the ^137^Cs inventory in the soil (unit: m^2^ kg^−1^)^18,54,55^. In 2015, when the impact of the initial ^137^Cs deposition would have been diminished, this ratio for oak trees was similar to, or greater than, that for cedar trees^55^. This result suggests that there is relatively active root uptake of ^137^Cs in konara oak. In addition, previous studies of decomposing litter and organic layer samples (see Table S2) have proposed that ^137^Cs exists in a more bioavailable form in konara oak than in Japanese cedar^32,44,50^; this result should lead to the relatively more active uptake of ^137^Cs by konara oak. Furthermore, fungal symbiosis might be associated with the differences in ^137^Cs accumulation from the soil between these two tree species. Generally, konara oak forms symbiotic associations with ectomycorrhizal species, whereas Japanese cedar forms associations with arbuscular mycorrhizal species. Incubation experiments on seedlings have suggested that the former fungal species enhance the solubilization of ^137^Cs-fixing minerals and root uptake of ^137^Cs to a greater degree than do the latter species^56–58^. However, not only variations in the impacts of the experimental conditions (e.g., the ^137^Cs and K concentrations and their ratio in the soil or solution culture) on the results^58–60^, but also completely opposite results—that is, a decline in root uptake^61^—have been reported. Further studies of the effects of fungal symbiosis on ^137^Cs accumulation are necessary.

Some of the ^137^Cs taken up by the roots is translocated to new leaves/needles and branches. Generally, the concentrations of K are higher in the leaves of deciduous broadleaf trees than in the needles of evergreen conifers, and soft leaves of deciduous broadleaf trees are relatively susceptible to leaching of K^62–64^. In the context of our comparison of the two species, this would be partly applicable ^137^Cs, and larger ^137^Cs fluxes from both tree to soil and soil to tree were estimated in konara oak trees. Our suggestion is comparable to the findings of Saidin et al. (2022)^26^. In 2017–2018, they found as much as 7.5 times greater tree-to-soil flux of ^137^Cs in konara oak stands than in Japanese cedar stands, despite similar initial deposition levels.

Finally, we also highlight the fact that ^137^Cs dynamics in forest ecosystems can be affected not only by the species but also by other environmental factors such as the topography and the quantity and quality of organic matter on the forest floor; moreover, these dynamics show large spatial variations^12^. This point has already been made by Koarashi et al. (2016) ^24^ in the initial phase after the accident. In addition to the species-specific ^137^Cs mobility examined in our experiments, further studies of such factors as well as continuous monitoring at multiple sites with a large sample size would enhance our understanding and forecasting of ^137^Cs dynamics in future decades.

## Conclusions

To (1) examine the mobility of ^137^Cs in different external parts of trees, and (2) compare ^137^Cs cycling in Japanese cedar and konara oak stands, we conducted leaching experiments on samples in ultrapure water or ammonium acetate. Our experiments revealed variations in the mobility of ^137^Cs in each part. For example, in Japanese cedar, the percentage of ^137^Cs leached from current-year needles ranged from 26 to 45% in ultrapure water and 27–60% in ammonium acetate and did not differ significantly from the percentages in old needles or in branches. In konara oak, the percentage of ^137^Cs leached from leaves was 47–72% in ultrapure water and 70–100% in ammonium acetate—similar to those from current-year and old branches. Markedly lower percentages of leached ^137^Cs were observed in the outer bark from Japanese cedar (0.70–19% in ultrapure water and 2.6–28% in ammonium acetate); this might have been caused by external contamination with insoluble particles bearing ^137^Cs. In addition, the percentage of ^137^Cs leached from the organic layer was extremely low—close to zero in ultrapure water—in both species. Overall, konara oak had greater ^137^Cs mobility than did Japanese cedar. From these results, as well as from our review of long-term monitoring studies and reports of active root uptake of ^137^Cs from the soil in konara oak, we suggest that more active cycling of ^137^Cs occurs in konara oak than in Japanese cedar. These findings provide some biochemical insights into spatially heterogeneous ^137^Cs cycling in forest ecosystems and its forecasting in the future.

## Supplementary Information

Supplementary Information 1.

## Data Availability

All data generated during this study are included in this article and its supplementary table.
